# Supplementary figures and images for: Movements of post-breeding royal terns (Thalasseus maximus) in Virginia, U.S.A
Source: PeerJ. 2025 Sep 18;13:e19898. doi: 10.7717/peerj.19898 (PMC12450368; doi:10.7717/peerj.19898)

step pseudo-residuals

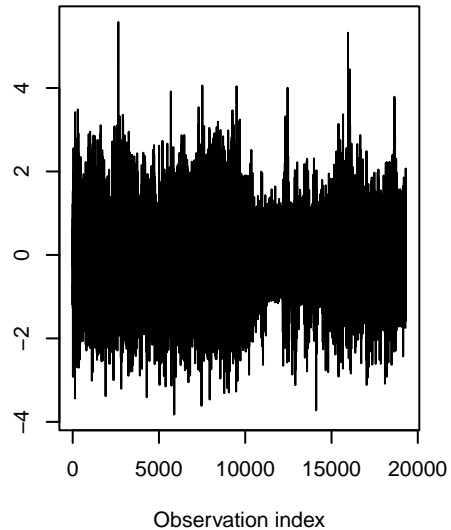

Sample Quantiles

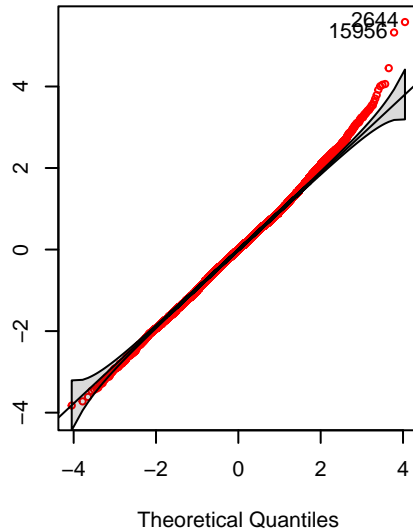

ACF

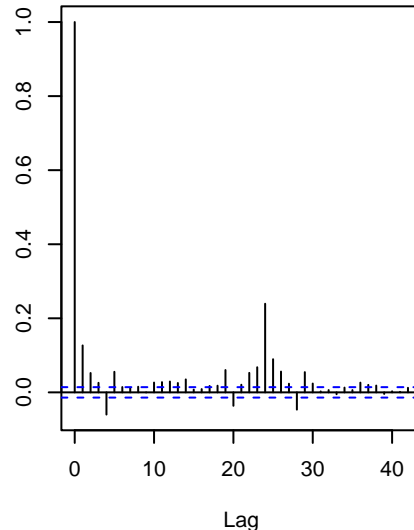

angle pseudo-residuals

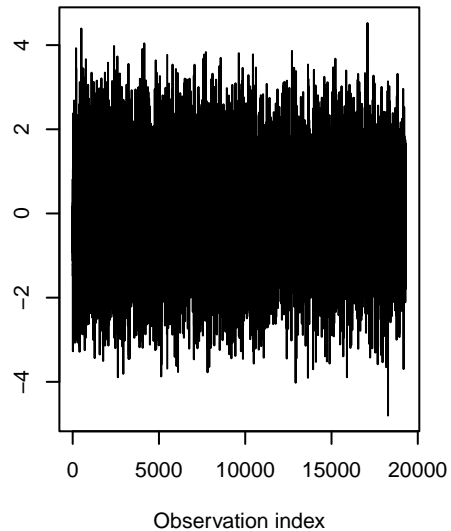

Sample Quantiles

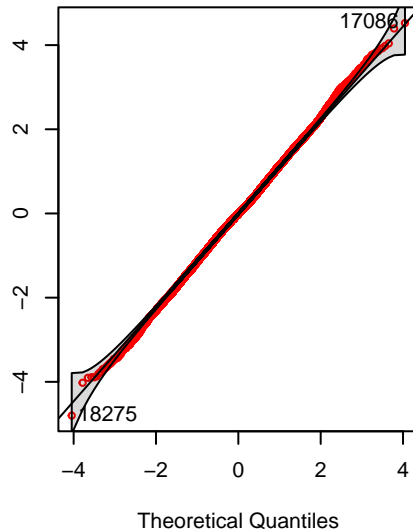

ACF

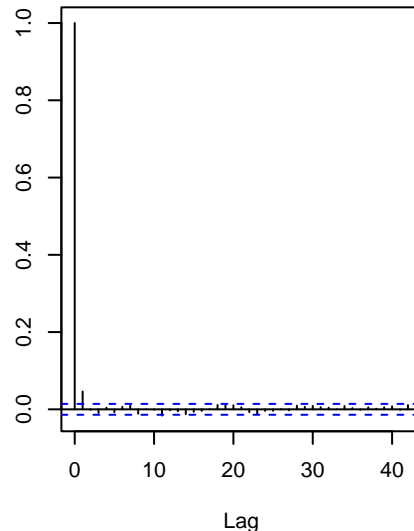

Supplement: Supplemental Information 2 [file peerj-13-19898-s002.pdf]

# All animals

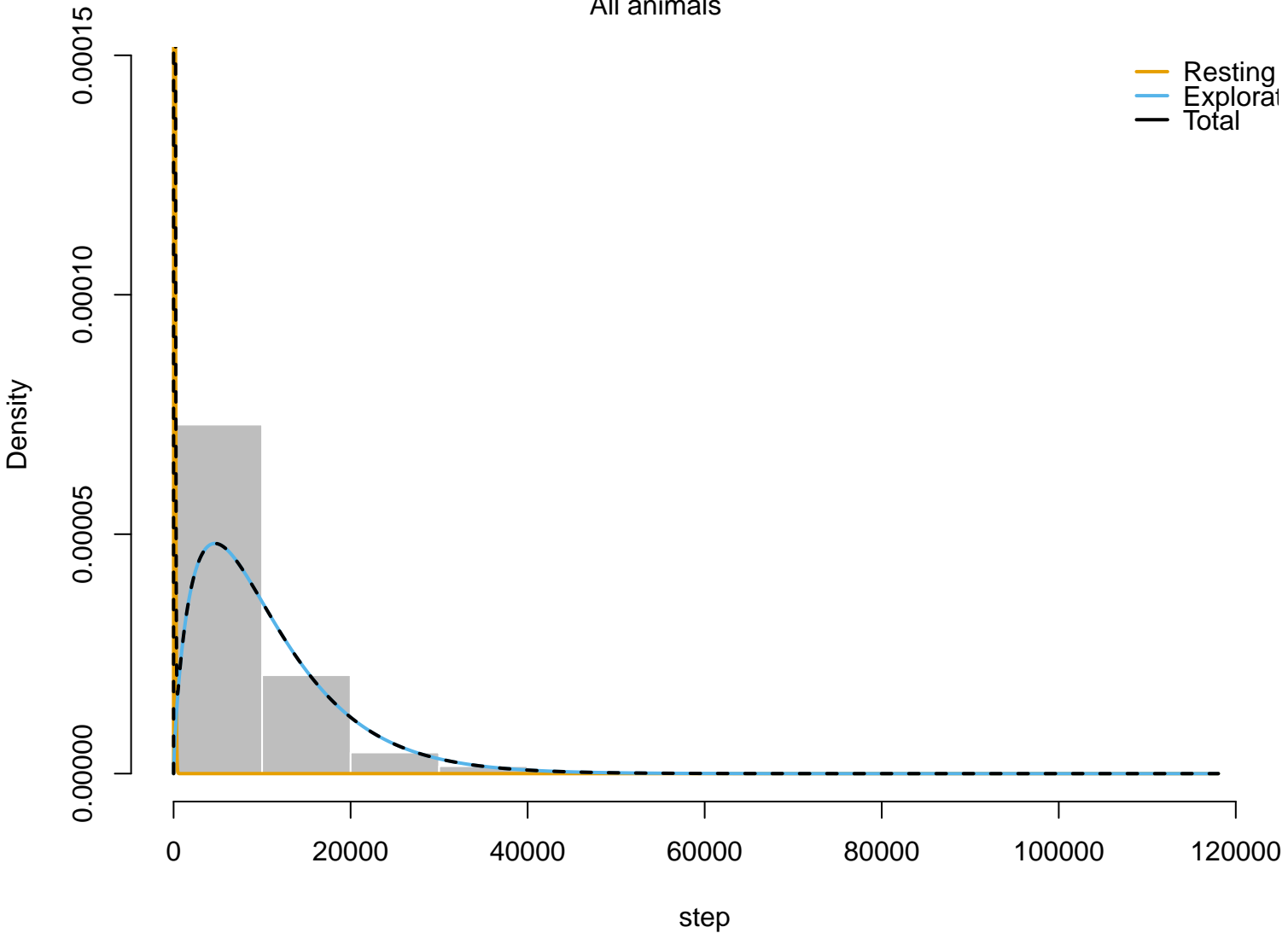

Supplement: Supplemental Information 3 [file peerj-13-19898-s003.pdf]

# All animals

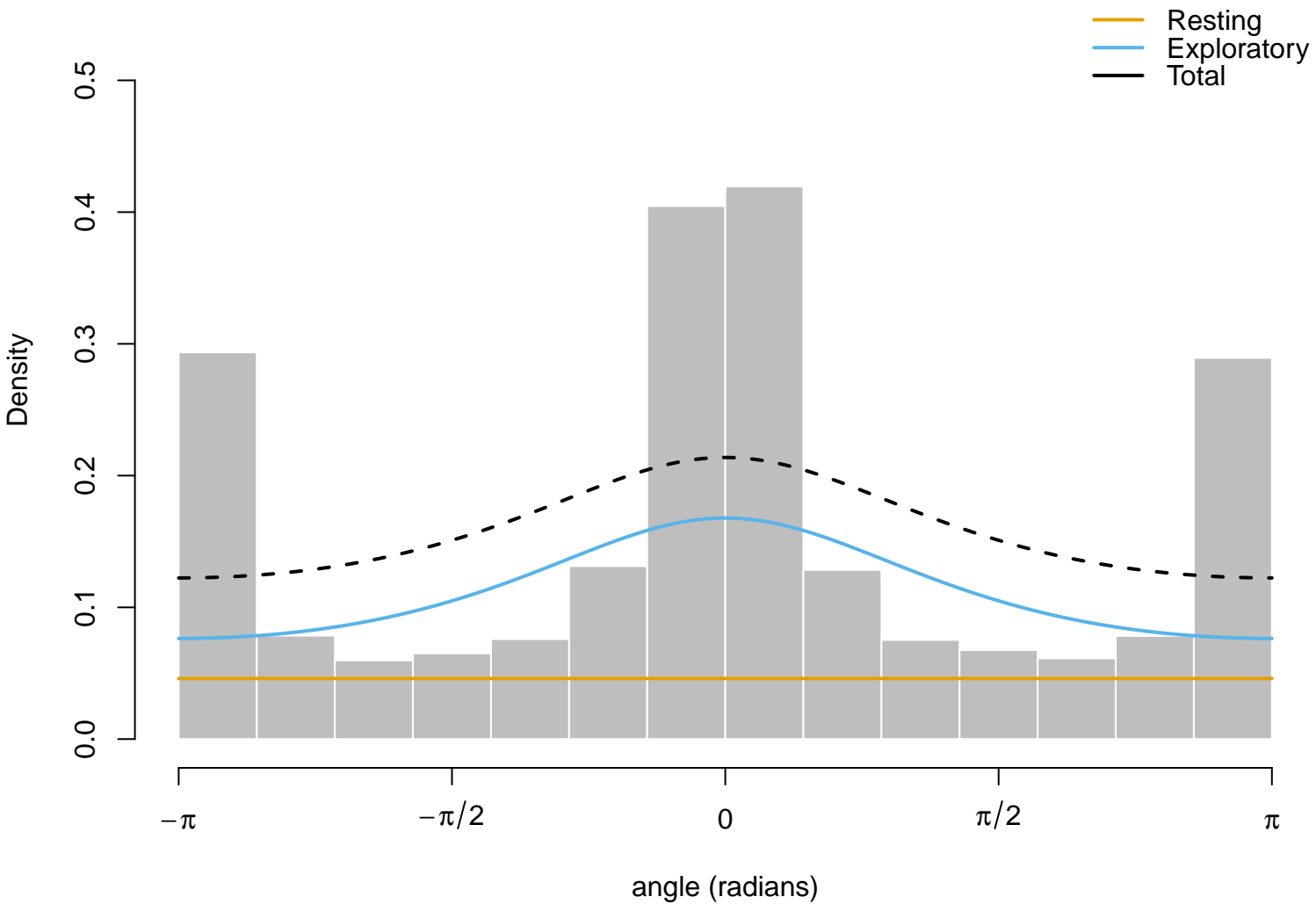

Supplement: Supplemental Information 4 [file peerj-13-19898-s004.pdf]
